# Illuminating the Psychological Experience of Elderly Loneliness from a Societal Perspective: A Qualitative Study of Alienation between Older People and Society

**DOI:** 10.3390/ijerph14070824

**Published:** 2017-07-21

**Authors:** Anna Wong, Anson K. C. Chau, Yang Fang, Jean Woo

**Affiliations:** 1 CUHK Jockey Club Institute of Ageing, The Chinese University of Hong Kong, Hong Kong, China; ansonchau@cuhk.edu.hk (A.K.C.C.); yang.fang@link.cuhk.edu.hk (Y.F.); jeanwoowong@cuhk.edu.hk (J.W.); 2 Department of Medicine & Therapeutics, The Chinese University of Hong Kong, Hong Kong, China

**Keywords:** loneliness, social exclusion, social isolation, urban living, aging, phenomenology

## Abstract

Loneliness is a common experience among older people that is associated with health risks and negative well-being. As a psychological phenomenon, it has typically been defined in Western research literature as the discrepancy between desired and actual interpersonal relations. In our qualitative study in Hong Kong, we offer insight into ageing and loneliness in an urban environment of the non-Western world and propose to reconceptualise loneliness by exploring older people’s experience of alienation at the societal level as an important but often neglected dimension of their loneliness. Thirty-seven community-dwelling, Chinese adults aged 65 and above were interviewed in focus groups and their accounts analysed and interpreted using a phenomenological approach. Findings revealed that focus group participants perceived insufficient care for older people, a growing distance between themselves and society, and their disintegrating identity in society to be primary sources of societal alienation. In response, older people adopted a more passive lifestyle, attributed marginalisation and inequality to old age, and developed negative feelings including unease towards ageing, vulnerability and helplessness, and anger. The emergence of these key components and underlying themes of societal alienation illuminated neglected facets of the psychological phenomenon of loneliness and highlighted new implications for policy, practice, and research from a societal perspective to address older people’s loneliness in urban settings.

## 1. Introduction

For more than 30 years, loneliness has been regarded as a ‘geriatric giant’ in the study of older people’s quality of life [1]. Research studies on its widespread and significant impact on health are continually being reported, culminating in a vast body of evidence that demonstrates negative effects of loneliness on the physical and mental health of older people, including higher risks of mortality [2,3,4,5,6], coronary heart disease and stroke [7], depression [8,9,10], cognitive decline and dementia [11,12,13,14].

Similarly, psychologists have long identified positive interpersonal relations as an essential component to feeling good and being well in life [15,16,17]. The importance of sociality continues into older age where leading a socially active life [18] and having accessible family ties, confidants, and close, harmonious personal ties [19,20] have positive effects on older adults’ well-being.

### 1.1. Existing Theories of Loneliness and Their Limitations

While many research studies on older people and loneliness have been preoccupied with associations between loneliness and its correlates, there has been less focus on theorizing the concept of loneliness itself. This gap is also demonstrated by most studies accepting from the outset that loneliness is defined by a deficit in interpersonal relationships, based on the widely used definition of ‘loneliness as a discrepancy between one’s desired and achieved levels of social relations’ [21] (p. 32). Yet, what constitutes social relations? Is loneliness experienced only as a result of deficits in interpersonal relationships?

As two of the few researchers who have made explicit the theoretical assumptions of their approach to studying loneliness, Peplau and Perlman [22] made a distinction between a social needs approach and a cognitive approach to conceptualizing loneliness based on attachment and cognitive evaluation theories, respectively. More recently, in a review by Victor et al. [23], four main theoretical perspectives were identified to be in common use, namely cognitive theory, which interprets loneliness as the negative outcome of the individual’s cognitive appraisal [22]; psychodynamic theory, which views loneliness as a pathology stemming from childhood experiences [24]; interactionist theory, which proposes that changes in loneliness are brought about by interacting conditions of the external social environment, the individual’s social network and his/her personal disposition [23]; and existential theory, which sees living with loneliness as mandatory for personal growth [25]. Apart from existential theory, current research on loneliness sets out to explain loneliness in terms of deficits in interpersonal relationships only, leaving the potential contribution of other relationships such as societal relations unaddressed. Some of the limitations of the aforementioned approaches will now be presented.

Firstly, as mentioned, the focus of a psychological inquiry is on the individual and his/her immediate social environment. By itself this does not address the related but distinct systems of sociology and philosophy [26], which have driven the studies of closely related concepts such as social exclusion [27,28], existential loneliness [25,29] and solitude [30,31]. Nonetheless, the study of loneliness as an immediate and emotional experience is well served by a psychological approach. Secondly, loneliness is itself a highly subjective experience, to which individuals attach meanings and interpretations that are driven by their expectations, values and beliefs [1]. These are both personal and shaped by the cultural ideologies that individuals have developed [32]. Therefore, a theoretical model that approximates loneliness more fully should also consider the contributing role of individuals’ subjective meaning-making as well as the cultural resources and context, especially with reference to different demographic groups. Thirdly, on a related note, initial research theories of loneliness have yet to be widely tested in non-Western settings and may not be wholly applicable, hence they warrant further investigation there.

### 1.2. Approaching Elderly Loneliness from a Societal Perspective in Non-Western Settings

Consequently, to address the first limitation, it would be helpful to broaden the psychological perspective and approach so that the study of loneliness may move beyond the interpersonal realm and out into the societal. For example, the philosopher Rubin Gotesky placed the experience of alienation at the heart of those concepts related to loneliness or ‘aloneness’ which evoke distressed emotions that may be experienced in relation to the society as well as to the self and to significant others [30]. In a series of experimental and correlational studies, Stillman et al. used chronic loneliness to reflect and measure an ongoing sense of social exclusion [33]. Addressing the other side of the same coin, Gibson looked at formation of meaningful societal connection as a way to overcome individual loneliness [34]. He used the examples of scientists and artists who spent a lifetime producing works that would earn them recognition and acceptance from the society with which they wished to form a meaningful connection [34], (p. 13); and of adolescents being heavily invested in the search for ‘meaning, identity and how they should relate to society’ [34], (p. 19). These examples also bring to mind the unique relevance that societal connection and alienation actually have for older people. On the one hand, older people have to confront inevitable life events such as retirement that would likely disrupt their existing connection with the society. On the other hand, they have to wrestle with establishing a new identity and role in society at a much later stage in life, which is all the more challenging.

Particularly in urban settings where retirees are in danger of feeling like a burden to the working population [35], to end older people’s employment purely due to chronological age is ‘to tell them that they are too old to be taken seriously in the world of affairs, that they have reached second childhood and that they should run away and play’ [36] (p. 38). This dismissiveness from society is effectively a ‘denial of the full rights of citizenship to some older people’, as it demands that they give up power in later life, lower their standard of living, and accept a less respected status in society once they reach a certain age ([36], pp. 36–37). The loss of ‘full citizenship’ described here through unwanted retirement not only affects the socioeconomic independence of older people, but also forces them to give up established social roles and identities that meaningfully connect them to society. This alienation from society, if left unaddressed, can turn into ostracism, which, Stillman et al. argued, was reflected in chronic loneliness and resulted in low meaning in life [33].

Therefore, a better understanding of older people’s loneliness may be achieved by considering their experiences of alienation at the societal level as well. In fact, Walsh et al. have developed a conceptual framework of ‘exclusion from social relations’, which is a useful tool for studying, discussing, and addressing complex societal issues affecting elderly people today [37]. However, to avoid confusion with sociological and political approaches to social exclusion that are informed by a more critical stance and focus on features of the external environment [38,39], this study has adopted the term ‘societal alienation’ to depict older people’s personal construal of their relationship with the society.

Secondly, in response to the role and contribution of individuals’ subjective meaning-making in loneliness, it would be useful to adopt an inductive and interpretive approach of enquiry that enables people’s experiences to emerge without the inhibition of predetermined responses and also captures the meanings that they personally assign to their experiences [40].

Thirdly, in response to the limited representativeness of current studies of loneliness among the older population in non-Western settings, we need to develop a more comprehensive and updated theoretical and empirical understanding of loneliness [1]. Current theories should therefore be continually tested, contextualised, and validated for older and non–Western populations in order to advance our understanding of loneliness and how it plays out for different demographic groups in different sociocultural settings.

### 1.3. The Context of Hong Kong: Unique Challenges in a Global City

The context of the current study on older people’s societal alienation is Hong Kong and it is recognised that certain tensions exist when examining the elderly’s quality of life living in the urban environment. On the one hand, efficient though arguably stretched structural support for the elderly. On the other hand, the economic, social and psychological well-being of older people is currently under a high level of threat. Although it is among the top countries and territories in terms of Gross National Income (GNI) per capita and indicators such as life expectancy at 60, physical safety, and access to public transport, Hong Kong’s elderly are faced with a high income disparity (based on its Gini coefficient of 0.533 in 2006), perceived inadequacy of retirement protection, old age poverty rate, relative welfare, and insufficient support for senior citizens to remain in employment. Furthermore, the economically developed territory consistently underachieved in indicators such as social connections and relative psychological well-being [41,42,43].

Taken together, these findings suggest that urban development does not necessarily go hand-in-hand with adequate protection of people’s well-being, especially the elderly. However, this critical issue is not unique to Hong Kong. As Cacioppo and Patrick [44] observed, it is a common phenomenon in many parts of the world and its root causes must be correctly identified and addressed. Therefore, this study would contribute towards understanding and addressing a global issue in similar urban contexts that are economically developed yet falling behind in terms of the well-being of the older population.

### 1.4. Alienation between Older People and Society

By drawing on Hong Kong as a theoretical case to study the phenomenon of alienation between older people and society, we aimed to extend our knowledge of older people’s loneliness beyond what has primarily been learned from Western settings. More importantly, however, through the present study we aimed to bring the less studied relationship between older people and the society into a reconceptualisation of loneliness among the older population. Such an approach would demonstrate the importance of examining alienation at the societal level of older people’s social worlds and take us beyond the notion that interpersonal relations are the primary dimension to be considered. Accordingly, an inductive and interpretive methodological approach was adopted to explore central themes that would emerge from first-hand, experiential data. Findings would also shed light on new ways to address loneliness among older people living in an urban environment, whether situated in Hong Kong or in similar socioeconomic settings. Thus, this study set out to answer the following question. What are the ways in which alienation at the societal level is experienced by older people living urban lives in Hong Kong?

## 2. Methods

To answer the research question, we set out to identify key components and underlying themes that represented alienation at the societal level from people’s subjective experiences of urban living in Hong Kong.

### 2.1. Design and Participants

Based on phenomenological assumptions, subjective meanings were sought through in-depth interviews. We employed a cross-sectional qualitative study design that enabled multiple perspectives to be sampled and examined alongside one another in the exploration of a new topic. Data were collected from four focus groups that comprised of 37 older people, 15 male and 22 female across two different age groups (aged 65 to 79 and aged 80 and above; for more details, see Table 1), to present a variety of perspectives from community-dwelling older people. Effort was made to obtain a balanced composition of age and gender within the group and this was usually the case except for the gender ratio in one group of elderly aged 80 and above, where male participants were very difficult to find or invite to the focus group. Some participants knew each other by face, others by first name, and some did not know each other. Participants were recruited through purposive sampling from four residential communities centred around public housing estates that operated under the Hong Kong government’s housing scheme for citizens who cannot afford private housing. According to the 2016 population by-census in Hong Kong [45], the percentages of the elderly population living in government-subsidised housing, i.e., public rental housing and subsidised home ownership housing are 36.7% and 19.2% respectively, while 42.8% lived in private permanent housing.

This study was carried out following the rules of the Declaration of Helsinki of 1975, revised in 2008. Research ethics approval was obtained from the Survey and Behavioural Research Ethics Committee (SBREC) of the Chinese University of Hong Kong. All focus group participants gave informed consent for participating in the research study and were compensated for their time with supermarket vouchers.

### 2.2. Data Collection

Four focus group interviews with nine or 10 participants in each were conducted within a two–month period. Each interview lasted around 90 min.

Participants were free to respond not only to the interviewer but to each other as they expressed their views from different perspectives. Hence, in addition to the interviewer’s prompts as one would in individual interviews, participants also stimulated each other, which often led to longer and richer discussions than anticipated. When polarisation of views emerged, the interviewer summarized what the opinions were and brought them to the attention of everyone in the group for further discussion until opinions were sufficiently expressed and debated without forcing them to resolve in the end. Lastly, group dynamics also played a role, mainly in affecting how many turns individual participants took. The interviewer was aware of this effect and carefully monitored the dynamics and made adjustments (such as asking quieter members to express their opinions) to minimize a minority of people dominating the group discussion. Having taken these steps, we found that group interviews did offer something distinctive from individual interviews because of the collective sense-making and co-construction of meaning among participants.

The questions were semi-structured, designed to explore older people’s experience of urban living. There are distinct disadvantages to asking participants about their loneliness directly [46]. The main methodological concern about explicitly asking about loneliness is that it may not elicit true responses from older people if they view loneliness as a stigmatizing experience. This study avoided such direct wording but instead asked participants to describe their experiences of urban living to begin with, encompassing both the positive and negative, followed by progressively focused questions (e.g., “Are you currently involved in some form of work, whether paid or unpaid?”, “Could you tell me more about your experience in it?” and “You have mentioned the difficulties of finding a job at this age. What are the main reasons for it?”). The discussion was guided through different aspects of urban living [47], such as affordability and accessibility of older people’s physical and social environments (e.g., housing, transportation, community resources) and perceived equality and attitudes towards the elderly in society, to ensure that multiple facets of urban living from a societal perspective had been considered. Then at the data analysis stage, themes of societal alienation were identified and extracted for analysis and interpretation.

### 2.3. Data Analysis and Interpretation

Guided by the phenomenological assumption that ‘human beings are not passive perceivers of an objective reality, but rather that they come to interpret and understand their world by formulating their own biographical stories into a form that makes sense to them’ [48] (p. 88), this study drew on the principles of Interpretive Phenomenological Analysis (IPA) to explore in depth participants’ meaning-making process, including their experience, understandings, perceptions and views [40]. This approach was particularly useful for exploring the current topic of societal alienation as experienced by older people in non–Western settings, because it enabled personal interpretations to come through, opening our eyes to their ‘thoughts, commitments and feelings through telling their own stories, in their own words, and in as much detail as possible’ ([40], p. 20).

Subsequently, descriptive accounts of urban living were thematically analysed in the following steps to reveal the fundamental ways in which societal alienation was experienced by older people amidst the physical and social environments in Hong Kong. Firstly, verbatim interview transcripts were coded to highlight participants’ subjective experiences of alienation in relation to the society. Open codes were then applied to these extracts to explore and describe the nature of participants’ experiences, such as the event they had perceived, how they had interpreted it and accompanying reasoning if provided, along with reports of surrounding conditions such as the contextual details and other people’s reaction. Through examining and comparing the initial open codes, categories were developed to summarise key components and underlying themes. A further stage of analysis involved seeking connections and identifying relationships between categories across the four datasets, rearranging them into levels from detailed to abstract, and constantly refining the emerging structure so that a final set of components, underlying themes and subthemes became representative of older people’s experience of alienation across the groups. This final representation, which includes participants’ quotes translated into English by the first author, who is bilingual, is presented in the following section.

## 3. Results

**Figure 1:**
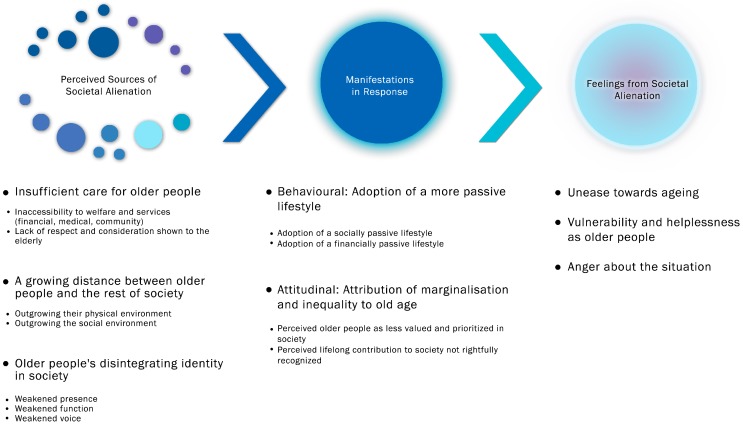
Key components and subthemes of older people’s experience of societal alienation.

Three main components of older people’s experience of societal alienation were identified, namely (i) perceived sources of societal alienation; (ii) manifestations in response; and (iii) feelings from societal alienation. Underlying themes for each component of the alienating experience are summarised in this section and graphically represented in Figure 1.

### 3.1. Perceived Sources of Societal Alienation

The analysis revealed three main sources of societal alienation as perceived by older people. These were *insufficient care for older people*, *a growing distance between older people and the rest of society*, and *older people’s disintegrating identity in society*.

#### 3.1.1. Insufficient Care for Older People

The perception of *insufficient care for older people* was first revealed in terms of inaccessibility to basic and well-deserved welfare and services, ranging from financial security and medical services to community resources. One of the major issues that emerged, especially among older people with low socioeconomic status (as indicated by living in public housing), was the lack of a satisfactory pension scheme for elderly people. Although already living in government-subsidised housing, some older people reported having to work well into their seventies in demanding jobs that did not pay well, e.g., in dressmaking factories and restaurants, in order to support themselves. Still, they were not able to save up a sufficient amount of money to have a sense of financial security. This seemed to be a common theme among the focus group participants, who had a lower socioeconomic status. While there was evidence of charity-led community initiatives that supported the less-well-off older people, focus group participants conveyed the feeling that it was socially less acceptable to ask for help, let alone subjecting oneself to formal financial assessment in order to qualify for free meals.

Access to medical services was another commonly cited difficulty for two reasons. Firstly, stretched resources at overcrowded hospitals meant that it was difficult for older people to access the medical services they needed:

*“If your case is not an emergency, say if something was wrong with my bone here, the doctor would put me on a waiting list for referral, and I would have to wait for two, three years. What happens is, I could walk now, but by the end of the three years of waiting, my leg would have gotten to the stage where I wouldn’t be able to walk”*.

*(Group B, aged 65–79)*

Secondly, the automated telephone booking system (formally the Hospital Authority General Out-patient Clinic Telephone Appointment System) currently in use in Hong Kong to arrange medical appointments for episodic illnesses such as influenza and gastroenteritis from home instead of queueing at the clinics, was notoriously inaccessible to older people:

*“Sometimes I don’t understand how it works. It is so difficult to book an appointment through the telephone booking system. Sometimes I think it is luck. If you are not as quick as the young people, if they are faster than you by a split second, then you would not be able to get through. Also, some of us don’t know what to do when asked to press one, then press two. When there are so many commands it is easy to press the wrong button. It’s impossible. Sometimes the automated phone even tells you in the first instance, ´we apologise but all places for medical appointment are now full´. That means we are not able to see a doctor”*.

*(Group D, aged 65–79)*

Participants also encountered insufficient community resources allocated to the elderly. Many reported a limited quota for joining physical or learning activities organised by the local government due to an insufficient number of venues and classes available. Some reported having to wait for as long as three years to be selected for a yoga or Tai Chi class, indicating a serious lack of provision for activity classes that are beneficial to the elderly’s health and well-being:

*“Health is most important to us now. We hope to be able to stay healthy through more exercising. It will help take pressure off the medical system too! But it is not possible even if we wanted to because of insufficient places. We hope that you can convey to the local government the need to expand the Sports Centre. Then we could have more classes and more participants can join”*.

*(Group D, aged 65–79)*

Apart from a lack of access to basic welfare and services, insufficient care for older people was also perceived in the general lack of respect and consideration shown to older people in everyday situations from being out and about in the neighbourhood and travelling on public transport, to eating out and doing grocery shopping, as the following quotes illustrate:

*“Talk about respect? They don’t even notice you because there is no exchange of communication. They just say ´hi´ to their phones all the time. Those people living opposite my flat won’t say good morning to me. They don’t even see me”*.

*(Group A, aged 80 and above)*

*“They won’t wait for you or give way to you. Elderly people walk more slowly, you know, much more slowly than youngsters. They don’t consider that. Bam! And they let the doors shut right in front of you. They won’t hold the lift for the elderly either. They press the ‘close the door’ button instead”*.

*(Group D, aged 65–79)*

Participants from one particular estate unanimously reported negligence and verbal abuse from staff and vendors who served and sold food there. Altogether, perceptions of inaccessibility to basic welfare and services and a general lack of respect and consideration formed the first main source of societal alienation in older people’s experience, which was insufficient care for them.

#### 3.1.2. A Growing Distance between Older People and the Rest of Society

The second source of alienation, *a growing distance between older people and the rest of society*, was a perception formed from their gradual awareness that as older people they were outgrowing their physical and social environment. This was especially heightened by their children’s generation having grown up and moved out of their neighbourhood, thus leaving them behind:

*“There are more and more elderly people in this housing estate. We were middle-aged when we first moved in and now have become elderly. This estate has become an elderly estate. The equipment was first built for our children when they were young, and thirty years have passed. We have become elderly and all the babies back then are now in their thirties and have moved out of the estate”*.

*(Group D, aged 65–79)*

When asked about their ideal place to live, one elderly person expressed the wish for estate planners to stop installing features that would remind them of being old and instead to bring in young people and children to liven up the place, further demonstrating a reluctance to be left behind and treated as a special, segregated demographic group. Yet, when there was a lack of attention paid to maintenance of their physical environment, the elderly also felt that they had outgrown their place, since neither its function nor usability had caught up with their ageing process. For example, the outdoor exercise area in one housing estate was installed over 20 years ago and still had not received any update, leaving the elderly to point out the inappropriateness of the balancing bars for their strength, while few children were around to use the play areas designated for them.

The notion that older people were outgrowing the rest of society was also explicitly expressed when discussing the automated telephone booking system, where they felt they were forced to catch up with the rest of society:

*“Regardless of who invented it, you have to adapt, there’s no other way from my point of view. Sure, the old system of queueing in person was better and easier to use, but times have changed and you have to adapt. If you don’t move with the times, the society will force you to anyway”*.

*(Group D, aged 65–79)*

Socially speaking, participants commonly cited age or the generational gap as an issue that affected them, as they described feeling distant from the young:

*“The young people nowadays are quite self-centred, maybe because they feel they are better educated than we are. There is some kind of distance between the young and us who are more elderly”*.

*(Group B, aged 65–79)*

For many, the perceived age gap definitely affected their willingness to interact with younger people. Behind the practical ways in which the ‘outdated’ housing estate, the new telephone booking system, or the age gap between old and young affected older people’s lives, there lies a deeper, more troubling sense of a widening gap between ‘them’ and ‘us—the older people’. The age-related distance was somehow unfavourable yet inevitable to them and little could be done about it. In older people’s view, they became the left-behind folks back home and slow-walking people on the street, and features that became increasingly unsuitable for use by older people received little attention or update even though the problems were known. As older people saw themselves outgrowing their physical and social environments, their feeling of being distant and alienated from the rest of the society also increased.

#### 3.1.3. Older People’s Disintegrating Identity in Society

The last source of alienation was revealed to be *older people’s disintegrating identity in society* due to their weakened presence, function, and voice. This was perhaps the most serious of the three sources of alienation because the fundamentals of who they were appeared to have been threatened as they entered the third and fourth ages. It was first expressed in terms of older people’s weakened presence, the loss or discontinuity of the physical and social self, in society. For example, the interviewed participants explicitly and implicitly cited age-related illnesses and mortality as responsible for the thinning effect on their generation:

*“When we were out and about in the last 10 years, we noticed that bunches of people have left or gone their separate ways. Some are not able to come out anymore and those who are left behind now are much smaller in numbers than before when we first retired. The group of us who used to come out between four and six in the afternoon, there just aren’t many of us now for some reason. Some can’t walk and others have left. They have all disappeared and it’s not surprising. After a certain age, say if you get a stroke, you will be gone”*.

*(Group B, aged 65–79)*

The loss of people of similar age affected their social selves too, as they saw themselves having weaker social connections and being more socially isolated. Gradually, they stopped engaging in activities that were once an important source of personal enjoyment and friendship. Participants contrasted the loss of social connections with a recollection of past liveliness down at the park, which used to be filled every Sunday by dozens of people, men and women, playing *Er-hu* (a popular Chinese stringed instrument) and singing Cantonese opera.

Secondly, their weakened function through reduced opportunity to work or contribute to society because of age was another reason that their identity was eroding away at the societal level. This had both financial and psychosocial implications. Although not representative of all retirees in Hong Kong, the participants saw their retirement as forced and felt reluctant about it. Some cited the unwillingness of employers to pay for their labour insurance as the primary reason why they could not hold onto their jobs:

*“Our chances are slim once past the age of sixty-five mainly because of labour insurance. It becomes expensive. The employer who wants to hire us would simply find it too expensive to pay for our insurance and in the end, decide against it”*.

*(Group D, aged 65–79)*

Others went on to express that these implicit rules that prevented them from work did not consider their real abilities and the need to function as before:

*“With all these standards dictating you, you just become an ‘elderly’ even if you don’t acknowledge being one. Many older people are physically very tough. Some of them are still the same physically and their intelligence has not declined. But if you put in these regulations then they can’t do anything about remaining in employment, except for civic or voluntary work. It’s simply not possible if you want work that pays you. So, an ‘elderly’ is someone defined by the age limitation that is set by the government”*.

*(Group D, aged 65–79)*

Many turned to voluntary work after retirement upon experiencing initial difficulties adjusting to life without work. However, volunteering at local community or elderly centres was not necessarily a solution for all. For example, some male retirees found it difficult to be in centres where the majority of the members were female.

Finally, older people’s weakened voice in society through often being neglected or pacified by people in power contributed to their disintegrating identity in society. Participants reported the rarity of being consulted by the local government on issues concerning their daily lives and community welfare even though they clearly expressed the desire to play a part in the community:

*“No one has invited us to talk about what’s going on in our local community. This interview is the first time. There are those who try to rally for our votes in local elections but none asked us to tell them what we thought of things like the community facilities, to be honest. We would want to participate and play a role in making the community better if we got the opportunity to do so”*.

*(Group D, aged 65–79)*

Some reported unhappy experiences that ended in quarrels or their views falling on deaf ears, describing the local politicians as ‘all liars’ who listened to their complaints but did not follow through:

*“There is consolation but no actual change. We talked to them and they said they would follow up. Yes, we would follow up, they said, but they never did. They are all liars. Talking about them makes me so cross”*.

*(Group C, aged 80 and above)*

In a discussion on the possibility of lowering the eligible age for medical vouchers, older people clearly felt that their voices would not count and that they had to prepare themselves for the government and local politicians not listening to them even if they spoke up:

*“Whatever our opinions are, we can raise them but there is no guarantee that they will be heard or accepted. Our guess is that the government has already prepared all these figures to show us why they will or will not do something. In the end, it would just be us talking and nothing further could happen”*.

*(Group D, aged 65–79)*

Therefore, findings have shown that as people entered the third and fourth ages, their identity in society was in danger of disintegrating through weakened presence, function, and voice, leading to a very serious form of societal alienation.

To sum up, analysis revealed three primary sources of societal alienation: *insufficient care for older people* in terms of inaccessibility to welfare and services and lack of respect and consideration shown to older people, *a growing distance between older people and the rest of society* as they found themselves outgrowing their physical and social environments, and finally their *disintegrating identity in society* through a weakened presence, function, and voice. Together these sources of societal alienation contributed to negative changes and feelings in older people’s lives, which are the other two key components in older people’s experience of societal alienation.

### 3.2. Manifestations in Response to Societal Alienation

According to older people’s accounts, societal alienation led to two main manifestations in response—behavioural and attitudinal—in the form of *adoption of a more passive lifestyle* and *attribution of marginalisation and inequality to old age*.

#### 3.2.1. Adoption of a More Passive Lifestyle

The behavioural manifestation, *adoption of a more passive lifestyle*, was revealed in two ways, firstly in older people’s social lives by increasing withdrawal from social interaction and activity in the community with people they did not know personally. For example, they described reducing interaction with others to avoid getting into potential situations of dispute:

*“It’s best not to get into quarrels. Just stop minding other people’s business and keep to yourself, as nothing good comes out of it. People around here are by and large friendly, so just remember to greet them and don’t do anything else, especially not get into quarrels with them”*.

*(Group A, aged 80 and above)*

In response to the poor treatment encountered in their local wet market, some reported going further afield to do their daily food shopping, whilst others resorted to quietly accepting their situation:

*“It’s fine by me. I just pay $2 (concessionary transportation fare) to travel to the next district to get my groceries done, where it is cheaper too. There’s just no need for all that trouble with the rude market vendor. I just go somewhere else. It’s simple and not worth going through all that trouble. Those who are able to and not wanting to quarrel with people can travel and shop elsewhere. But those not able to will have to stay behind”*.

*(Group C, aged 80 and above)*

Their passive social lifestyle was also demonstrated in reports of a strong reliance on local elderly centres run by Non-Governmental Organisations to maintain their sense of purpose in daily lives, and without which they immediately felt substantial emptiness. More importantly, interviews revealed that this form of community engagement tended to be top–down, whereby members simply turned up to be entertained instead of taking charge of the mode of engagement.

Secondly, the adoption of a more passive lifestyle was related to older people’s financial situation post-retirement. Those who were out of work and financially dependent exercised limited choices in their daily lives because of reduced income. In particular, it was found that when they were aged 60 to 64, costs of essential services such as transportation and healthcare were at the forefront of their minds. They labelled this financially tight period the ‘vacuum period’ which falls between the end of their paid employment and the beginning of their eligibility for elderly benefits:

*“It’s tough for those not yet reaching the age of 65. Sometimes you have to stop and think before travelling over to the Island side* [The far side of town for the participants who lived in New Territories, Hong Kong] *because you have no income during this vacuum period. It is an awkward age and transportation costs are really expensive. Therefore, those in their early 60s can’t travel as frequently and have to endure the situation for a few more years before paying the concessionary fare ($2). We know friends who went out for tea with others and the cost per head worked out to be less than the individual’s transportation fee. It’s a common problem all over Hong Kong. However, it is really worth considering other solutions, since the government effectively makes you retire at age 60, not 65, so concessions should not start at age 65. Even a slight discount earlier would be good for this period of zero income”*.

*(Group D, aged 65–79)*

*“Some people really can’t afford to spend money on advanced services offered by privately run elderly centres. Some elderly have children who are filial and will see their parents on festive days and give them money every month. If this wasn’t the case then the elderly would have to rely on the government’s support. They get about 2000 dollars* [around 250 U.S. dollars] *each month for basic living expenses, and they just cannot afford to go to centres which offer better services”*.

*(Group B, aged 65–79)*

In sum, as people got older, they not only became more passive socially, but also financially. Particularly, those with low socioeconomic status tended to be on the receiving end of financial support perceived as insufficient, which in turn reinforced their adoption of a passive lifestyle overall.

#### 3.2.2. Attribution of Marginalisation and Inequality to Old Age

The second, attitudinal, manifestation in response to societal alienation was found in older people’s *attribution of marginalisation and inequality to old age*. As older people encountered increasing difficulties accessing or receiving essential welfare and services, they began to attribute them to ageist tendencies in society as a whole. Participants expressed a general sense that older people are not as valued and prioritised in society, for example when it came to the allocation of community space and facilities. They perceived that the local government had not considered nor actively supported older people’s needs and willingness to engage in social activities or health-promoting exercises. Instead, older people felt they were left to their own devices to fight with other demographic groups for community resources.

Still others felt that their contribution to society when they were younger and economically active had not been rightfully recognised once they became older and retired members of society. Specifically, they felt that many in financial need were not looked after by the elderly benefits system:

*“For many people, the system means that they would not qualify for the elderly benefit. In my case, my household only just qualified after jointly applying with my husband who is older and had been without income for some time. If I lived alone, I would not qualify even though I worked hard all my life here in Hong Kong”*.

*(Group C, aged 80 and above)*

In summary, analysis revealed that in response to societal alienation, older people *attributed perceived marginalisation and inequality to old age*, forming the interpretations that older people were not as valued or prioritised as other demographic groups, and that their lifelong contribution to society had not been rightfully recognised. Together with *the adoption of a more passive lifestyle*, older people were found to have manifested behavioural and attitudinal changes in response to societal alienation.

### 3.3. Feelings from Societal Alienation

In our analysis, we found that older people expressed a wide range of feelings as they described encountering alienation in the society. This included *unease towards ageing*, *vulnerability and helplessness as older people*, and for some, *anger about their situation*.

First of all, the feeling of unease towards ageing sprang from the awareness that their generation of people was becoming increasingly isolated from the rest of society and gradually disappearing due to inevitable age-related illnesses or death. For example, contrasting use of words and phrases was found in participants’ description of the past and present states of their community. This was interpreted as revealing their negative feelings on being an older group or community. When reminiscing about the past, they used “together”, “full”, “play”, “however we wanted (i.e., choice)” and mentioned large numbers of people and a variety of activities these people engaged in; whereas referring to the present, they switched to words or phrases like “difficult”, “dispersed”, “a few here and a few there”, “three or four kittens” (idiom for “few people showing up”), and “uncertain” in the immediate context of describing the state of the same community after folks disappeared, had a stroke, had restricted mobility, or were housebound. The sense of unease also emerged when they referred to disadvantaged or undesirable situations in which they found themselves due to old age and when they put a label on themselves by calling their housing estate ‘old people’s village’ with a negative connotation.

Secondly, feelings of vulnerability came from a sense of not being well protected in a society where caring and protective individuals were reportedly rare and the people they frequently met tended to focus on the self above the welfare and needs of older people, thereby putting them in a disadvantaged position. For example, they described being anxious and hypervigilant when walking down the street. Some encountered abusive behaviour because they were easy targets who could not fight back. Others felt that they were treated with neglect as invisible or forgotten people. Added to this, a feeling of helplessness also came with the passive lifestyle they took on as their identity in society gradually disintegrated and they perceived themselves as counting for less financially, socially, and civically. For example, they described feeling lost when they did not have access to the elderly community centre for support temporarily. Consequently, they were less vocal in speaking up for their rights and were left to fend for themselves with limited resources, resulting in a sense of helplessness.

Some elderly participants expressed anger at the ways they felt they had been treated by society especially in terms of the lack of well-deserved financial protection for older people and the pacifying attempts to quiet their complaints but not follow them up effectively if at all. Even though most had expressed a desire for societal change when discussing issues that alienated them as older people, they simultaneously conveyed a stoic acceptance of very little chance of such change taking place. In summary, these negative feelings were found to be implicit in participants’ response and interpreted as feelings resulting from societal alienation. The ways in which they related to loneliness will be discussed in the next section.

## 4. Discussion

By studying the experiences of alienation at the societal level among older people in Hong Kong, this study has extended current knowledge and conceptualisation of their loneliness in three main ways. Firstly, in line with conclusions from studies of loneliness and social exclusion in the United Kingdom [49], Hong Kong elderly’s experience of alienation included clearly identifiable societal triggers, or ‘structural drivers’, of loneliness from the society’s insufficient care for older people, through the perception of a growing distance between older people and the rest of society, to the sense that their identity in society was disintegrating, challenging their very existence and value as society members once they entered into old age. Importantly, findings suggested that older people who were interviewed had internalised perceptions of the society’s alienation towards them, resulting in behavioural and attitudinal changes that further reinforced their situation in a vicious cycle. Subsequently, they felt uneasy, vulnerable, helpless, and even angry as a social group alienated for growing old. These findings began to resonate with ‘ethical loneliness’, a type of loneliness articulated by Stauffer in the following way:

“Ethical loneliness is the isolation one feels when one, as a violated person or as one member of a persecuted group, has been abandoned by humanity, or by those who have power over one’s life’s possibilities. It is a condition undergone by persons who have been unjustly treated and dehumanized by human beings and political structures, who emerge from that injustice only to find that the surrounding world will not listen to or cannot properly hear their testimony—their claims about what they suffered and about what is now owed them—on their own terms. So ethical loneliness is the experience of having been abandoned by humanity compounded by the experience of not being heard” [50], (p. 1).

Indeed, the elderly living in a socioeconomic context like Hong Kong face challenges that are not necessarily encountered in Western societies where a well-developed system of pensions and welfare for the elderly is in place. According to a recent global comparison of older people’s health, economic, and psychosocial well-being in 97 countries and territories [41], Hong Kong rated quite poorly in the domain of Income Security compared to its Western counterparts and for such an economically developed territory. Particularly, Hong Kong’s rankings in the indicators Pension Coverage and Poverty Rate are 60 and 94 out of 97, respectively. Despite having one of the highest GNI per capita in the world, the combination of Hong Kong’s high living costs and the lack of a comprehensive welfare system have made it a particularly challenging place for poorer and socially vulnerable elderly people to grow old in. Coupled with the unlikelihood of the elderly speaking up and being heard, ageing can be a very alienating experience for the average Hong Kong elderly.

Secondly, having identified other important facets of ‘discrepancy between desired and actual social relations’ [21], (p. 32) that must be addressed at the societal level, this study has made a case for expanding future research on loneliness beyond the interpersonal level to consider people’s relationship to society as well. Our findings demonstrate that older people’s perceptions and feelings from societal alienation echo those items from the most widely used University of California Los Angeles (UCLA)-Loneliness Scale (Version 3) [51], such as ‘There is no one I can turn to’, ‘I am no longer close to anyone’, and ‘I feel left out’, suggesting that societal connection and alienation are closely related to individuals’ subjective experience of loneliness. Further, in a recent review of loneliness research, de Jong Gierveld et al. outlined macro-level factors that influence personal loneliness, such as demographic composition, cultural norms and values, and societal welfare, and the direct and indirect pathways by which societal inequality can affect well-being and loneliness [52]. Informed by findings from the current study and latest research development, this study thus proposes to expand the definition of Peplau and Perlman’s ‘social relations’ [21], (p. 32) beyond the interpersonal level to better represent the complex interacting systems that constitute older people’s social worlds in which causes and experiences of loneliness may be found and intervened at multiple levels and domains [36,37,44]. By broadening the definition of loneliness to study the experience of alienation and the feeling of being abandoned, rejected, and the associated pain, this study joined others in exploring these broader concepts in studies of loneliness [53,54,55,56].

Thirdly, our initial findings show that causes perceived by the Hong Kong elderly as negatively affecting them fit well into the social exclusion framework developed by Walsh et al. [37]. Yet, we also see the importance of further investigating the nature of loneliness as a subjective feeling arising from an array of factors both interpersonal and societal. Therefore, our study adopted a phenomenological approach to capture elderly people’s subjective experience of urban living, producing further findings that not only informed us of perceived causes of alienation, but of elderly’s behavioural and attitudinal response as well as their feelings. Using an inductive and interpretive methodological approach allowed this study to explore older people’s subjective meaning-making of their societal alienation in urban Hong Kong, a previously less studied area. In doing so, this study avoided the pitfall of predetermining the outcomes of participants’ responses, and extended our knowledge of older people’s experiences beyond Western settings. Also, such an approach would connect us with the psychological literature of elderly’s negative emotions and psychological well-being as well as the wider literature on associations between loneliness and health risks outlined in the introduction of the paper, thereby laying the groundwork for further investigation into this phenomenon and its implications.

### 4.1. Practical Implications

At a local and practical level, findings have revealed the need for the government and policymakers to critically address ways in which urban living can make older people feel alienated and for them to strengthen areas where meaningful connection may be restored, thus reducing older people’s loneliness. Insight from this exploratory study suggests directions in which this may be achieved. First of all, a change of perception towards how older people and the society relate to each other is a prerequisite. There must not be a deep, alienating divide between ‘us’ and ‘them’ to begin with. Instead, older people should feel that they are related to as part of the same fabric that all members come from and depend upon for a cohesive society. This change necessitates older people’s views and needs to be truly valued, represented, and understood from their perspectives as well as in the bigger picture by all members of society. The resulting deep listening and genuine conviction to follow up their expression is what older people conveyed that they wanted, especially from those who are in power and influential on their lives on a large scale. This brings us to the second point. In accordance with recent research findings in Hong Kong [57], it is specifically the welfare and pension systems, the provision and allocation of health and community resources, and the creation of alternative solutions to keep older people in employment if desired that have been identified as critically affecting older people’s experience of alienation vs. connection. This study thus argues that these sources of societal alienation must be addressed to prevent older people from living and feeling like the lonely, left-behind generation.

To this end, an age-friendly community perspective has been proposed [58,59] for identifying social and physical components of urban neighbourhoods that may be advantageous or barriers to reducing loneliness and social isolation among older people. In Canada, for example, loneliness was selected as one of four indicators for evaluating health and social outcomes of age-friendly communities [60]. In Hong Kong, the Age-Friendly Communities initiative has recently begun to filter down from the central government to the local district councils as a priority area to pour resources into [61]. The Age-Friendly City framework developed by the World Health Organization (WHO) evaluates age-friendliness in urban settings and encourages both government-supported and community-driven approaches to bring about societal changes that would improve older people’s quality of life [62]. Evaluations in Hong Kong are currently underway to assess a comprehensive range of physical and social environmental features that will influence elderly’s quality of life. It is expected that they would continually inform community stakeholders including local governors and community partners in how they address the problems raised by their community members. Initiatives like this would certainly help identify sources of alienation and articulate issues that require action in order to reduce the chronic loneliness experienced by older people in relation to their society.

### 4.2. Limitations and Future Research

Some limitations of this study were considered. Firstly, the current study aimed to address societal alienation as experienced by older people and in doing so we deliberately focused on effects of societal attributes without further investigation into both interpersonal and societal factors and how they separately and together affect elderly’s feelings of loneliness, we do not claim that the role of societal alienation is the most pertinent in the study of elderly’s loneliness. We do, however, find that it is an aspect worth highlighting and investigating further in the elderly population particularly, where increasing dependence on the welfare system, healthcare services, and community support can increase the elderly’s vulnerability to perceived isolation and alienation, resulting in heightened feelings of loneliness.

Secondly, socioeconomic status has been found to be associated with loneliness [63]. A caveat of this study lies in its sampling of residents of public housing estates and hence the socioeconomically more disadvantaged elderly in Hong Kong. Future studies into elderly loneliness should include the less disadvantaged elderly and explore any additional themes of societal alienation in that subgroup.

Thirdly, as put forward by Bronfenbrenners’ ecological systems theory [64], influences between systems in a social world are bidirectional. For example, cultural attitudes towards ageing, which belong to the macrosystem, will influence community and health services and mass media in the following mesosystem, and vice versa. Therefore, having identified the societal dimension of the loneliness experience, future research should study comprehensively the ways in which societal alienation might be transmitted or buffered through community and interpersonal components in the meso- and microsystems before affecting loneliness at the individual level. This valuable work, which is already underway in social exclusion research [37] and Western settings [65], currently awaits further study in non-Western contexts. Future studies can build on existing findings to evaluate both objective factors leading to social exclusion using the aforementioned framework and subjective feelings and perceptions that lead to the feeling of loneliness, and explore their associations. As more facets and contexts of loneliness are explored and the relationships among them examined, older people’s loneliness may be captured in a more differentiated and meaningful model that represents their psychosocial worlds even more closely. Also, in addition to the theoretical or analytic generalisation achieved in qualitative research, it would be important in future research to quantify the experience of societal alienation in order to achieve empirical generalisation as well [66,67].

Finally, it was outside the scope of this study to investigate important components and factors underlying loneliness at the intrapersonal level, such as solitude [30,31,68], meaning in life [25,29,33], and attitude towards imminent death [69,70,71], which all have a role to play in older people’s experience of loneliness. Therefore, future research in the field of ageing would benefit from drawing on perspectives in philosophy and existential psychology to examine loneliness among the older population.

## 5. Conclusions

This study has extended current conceptualisation of elderly loneliness by using a cross-sectional qualitative study to demonstrate the importance of a societal perspective to illuminate previously neglected aspects of the psychological experience. Rather than leaving the elderly to withdraw to a passive lifestyle and feel uneasy, vulnerable, helpless and angry about their current situation, findings from this study suggest that loneliness may be reduced by addressing the identified sources of societal alienation and increasing the support and empowerment of older people in society so that they can proactively engage in improving quality of life for themselves and for all older people. The kind of societal changes advocated in this study are vital if we wish to see improvement in older people’s well-being in Hong Kong, and indeed other similar settings, until the benefits of the city’s urban development finally outweigh its harms for this population. Most importantly, the pain of alienation should no longer be experienced by our society’s longest-serving members. Instead, through sustained efforts to increase society’s understanding of older people’s loneliness and restoring connections at multiple levels and domains, we hope that older people can reclaim the experience of being respected, valued, and wanted members of society.

## Figures and Tables

**Table 1:** Demographic description of focus group participants by age group, gender, marital status, living arrangement, and employment status.

Demographic Description	*n* (%)
Age group	
Aged 65–79	19 (51.4%)
Aged 80 and above	18 (48.6%)
Gender	
Female	22 (59.5%)
Male	15 (40.5%)
Marital Status	
Married	28 (75.7%)
Widowed	4 (10.8%)
Never married	1 (2.7%)
Unknown	4 (10.8%)
Living Arrangement	
Living with spouse and/or family	30 (81.1%)
Living alone	4 (10.8%)
Unknown	3 (8.1%)
Employment Status	
Working (in paid employment)	1 (2.7%)
Not working	36 (97.3%)
Total Number	37

## References

[1] Routasalo P., Pitkala K.H. (2003). Loneliness among older people. Rev. Clin. Gerontol..

[2] Chan A., Raman P., Ma S., Malhotra R. (2015). Loneliness and all-cause mortality in community-dwelling elderly Singaporeans. Demogr. Res..

[3] Holt-Lunstad J., Smith T.B., Baker M., Harris T., Stephenson D. (2015). Loneliness and Social Isolation as Risk Factors for Mortality: A Meta-Analytic Review. Perspect. Psychol. Sci..

[4] Holwerda T.J., Beekman A.T.F., Deeg D.J.H., Stek M.L., van Tilburg T.G., Visser P.J., Schmand B., Jonker C., Schoevers R.A. (2012). Increased risk of mortality associated with social isolation in older men: Only when feeling lonely? Results from the Amsterdam Study of the Elderly (AMSTEL). Psychol. Med..

[5] Holwerda T.J., van Tilburg T.G., Deeg D.J.H., Schutter N., Dekker J., Stek M.L., Beekman A.T.F., Schoevers R.A., Rien V. (2016). Impact of loneliness and depression on mortality: Results from the longitudinal ageing study Amsterdam. Br. J. Psychiatry.

[6] Shiovitz-Ezra S., Ayalon L. (2010). Situational versus chronic loneliness as risk factors for all-cause mortality. Int. Psychogeriatr..

[7] Valtorta N.K., Kanaan M., Gilbody S., Ronzi S., Hanratty B. (2016). Loneliness and social isolation as risk factors for coronary heart disease and stroke: Systematic review and meta-analysis of longitudinal observational studies. Heart.

[8] Adams K.B., Sanders S., Auth E.A. (2004). Loneliness and depression in independent living retirement communities: Risk and resilience factors. Aging Ment. Health.

[9] Cacioppo J.T., Hughes M.E., Waite L.J., Hawkley L.C., Thisted R.A. (2006). Loneliness as a specific risk factor for depressive symptoms: Cross-sectional and longitudinal analyses. Psychol. Aging.

[10] Cacioppo J.T., Hawkley L.C., Thisted R.A. (2010). Perceived social isolation makes me sad: 5-year cross–lagged analyses of loneliness and depressive symptomatology in the Chicago Health, Aging, and Social Relations Study. Psychol. Aging.

[11] Zhong B.L., Chen S.L., Conwell Y. (2016). Effects of transient versus chronic loneliness on cognitive function in older adults: Findings from the Chinese Longitudinal Healthy Longevity Survey. Am. J. Geriatr. Psychiatry.

[12] Holwerda T.J., Deeg D.J.H., Beekman A.T.F., van Tilburg T.G., Stek M.L., Jonker C., Schoevers R.A. (2014). Feelings of loneliness, but not social isolation, predict dementia onset: Results from the Amsterdam Study of the Elderly (AMSTEL). J. Neurol. Neurosurg. Psychiatry.

[13] Tilvis R.S., Ka M.H., Jolkkonen J., Valvanne J. (2004). Predictors of Cognitive Decline and Mortality of Aged People over a 10-Year Period. J. Gerontol. A Biol. Sci. Med. Sci..

[14] Wilson R.A., Frueger K.R., Arnold S.E., Schneider J.A., Kelly J.F., Barnes L.L., Tang Y., Bennett D.A. (2007). Loneliness and risk of Alzheimer disease. Arch. Gen. Psychiatry.

[15] Baumeister R.F., Leary M.R. (1995). The need to belong: Desire for interpersonal attachments as a fundamental human motivation. Psychol. Bull..

[16] Ryff C.D., Keyes C.L. (1995). The structure of psychological well–being revisited. J. Pers. Soc. Psychol..

[17] Rowe J.W., Kahn R.L. (2015). Successful Aging 2.0: Conceptual Expansions for the 21st Century. J. Gerontol. Ser. B Psychol. Sci. Soc. Sci..

[18] Gerstorf D., Hoppmann C.A., Löckenhoff C.E., Infurna F.J., Schupp J., Wagner G.G., Ram N. (2016). Terminal decline in well–being: The role of social orientation. Psychol. Aging.

[19] Litwin H., Stoeckel K.J. (2014). Confidant network types and well-being among older Europeans. Gerontologist.

[20] Rook K.S., Mavandadi S., Sorkin D.H., Zettel L.A. (2007). Optimizing social relationships as a resource for health and well-being in later life. Handbook of Health Psychology and Aging.

[21] Perlman D., Peplau L. (1981). Toward a social psychology of loneliness. Personal Relationships in Disorder.

[22] Peplau L.A., Perlman D. (1982). Perspectives on loneliness. Loneliness: A Sourcebook of Current Theory, Research and Therapy.

[23] Victor C., Scambler S., Bond J., Bowling A. (2000). Being alone in later life: Loneliness, social isolation and living alone. Rev. Clin. Gerontol..

[24] Fromm-Reichmann F. (1959). Loneliness. Psychiatry J. Study Interpers. Process..

[25] McGraw J.G. (1995). Loneliness, its nature and forms: An existential perspective. Man World.

[26] Mijuskovic B.L. (2012). The sociology and psychology of loneliness. Loneliness in Philosophy, Psychology, and Literature.

[27] Mathieson J., Popay J., Enoch E., Escorel S., Hernandez M. (2008). Social Exclusion: Meaning, Measurement and Experience and Links to Health Inequalities: A Review of Literature.

[28] Silver H.F. (2007). The Process of Social Exclusion: The Dynamics of an Evolving Concept.

[29] Moustakas C.E. (2016). Loneliness. http://pp-publishing.com.

[30] Gotesky R., Edie J.M. (1965). Aloneness, loneliness, isolation, solitude. An Invitation to Phenomenology.

[31] Neale R.E. (1984). Loneliness, Solitude, Companionship.

[32] Cole M. (1998). Cultural Psychology: A Once and Future Discipline.

[33] Stillman T.F., Baumeister R.F., Lambert N.M., Crescioni A.W., DeWall C.N., Fincham F.D. (2009). Alone and without purpose: Life loses meaning following social exclusion. J. Exp. Soc. Psychol..

[34] Gibson H.B. (2000). What is loneliness?. Loneliness in Later Life.

[35] Wells N.E., Freer C. (1988). The Ageing Population: Burden or Challenge?.

[36] Gibson H.B. (2000). The problems of later life. Loneliness in Later Life.

[37] Walsh K., Scharf T., Keating N. (2016). Social exclusion of older persons: A scoping review and conceptual framework. Eur. J. Ageing.

[38] Townsend P. (1979). Poverty in the United Kingdom: A Survey of Household Resources and Standards of Living.

[39] Levitas R., Pantazis C., Fahmy E., Gordon D., Lloyd E., Patsios D. (2007). The Multi-Dimensional Analysis of Social Exclusion.

[40] Reid K., Flowers P., Larkin M. (2005). Exploring lived experience: An introduction to interpretive phenomenological analysis. Psychologist.

[41] The Chinese University Hong Kong Jockey Club Institute of Ageing (2017). Report on AgeWatch Index for Hong Kong 2015.

[42] The Chinese University of Hong Kong Survey Findings on Views on Retirement Protection in Hong Kong Released by Hong Kong Institute of Asia-Pacific Studies at CUHK. http://cpr.cuhk.edu.hk/en/press_detail.php?id=2183&t=survey-findings-on-views-on-retirement-protection-in-hong-kong-released-by-hong-kong-institute-of-asia-pacific-studies-at-cuhk.

[43] The University of Hong Kong Public Opinion Programme Survey on Retirement Plan and MPF 2016. https://www.hkupop.hku.hk/english/report/MPF2016_Convoy/index.html.

[44] Cacioppo J.T., Patrick W. (2008). The power of social connection. Loneliness: Human Nature and the Need for Social Connection.

[45] Census and Statistics Department of Hong Kong Special Administrative Region Government (2017). 2016 Population By-Census: Population in Domestic Households by Sex, Age, Year and Type of Housing.

[46] Victor C., Grenade L., Boldy D. (2005). Measuring loneliness in later life: A comparison of differing measures. Rev. Clin. Gerontol..

[47] World Health Organization (2015). Measuring the Age-friendliness of Cities: A Guide to Using Core Indicators.

[48] Brocki J.M., Wearden A.J. (2006). A critical evaluation of the use of interpretative phenomenological analysis (IPA) in health psychology. Psychol. Health.

[49] Scharf T. (2012). Loneliness and Social Exclusion: Understanding Risks and Influences. MICRA Ageing Loneliness Semin.

[50] Stauffer J. (2015). Ethical Loneliness: The Justice of Not Being Heard.

[51] Smith J.M. (2012). Toward a better understanding of loneliness in community-dwelling older adults. J. Psychol..

[52] De Jong-Gierveld J., van Tilburg T., Dykstra P.A., Vangelisti A., Perlman D. (2016). Loneliness and social isolation. Cambridge Handbook of Personal Relationships.

[53] Eisold K., Willock B., Bohm L.C., Curtis R.C. (2012). The threat of exile and abandonment. Loneliness and Longing: Conscious and Unconscious Aspects.

[54] Willock B., Willock B., Bohm L.C., Curtis R.C. (2012). Loneliness, longing and limiting theoretical frameworks. Loneliness and Longing: Conscious and Unconscious Aspects.

[55] Rokach A. (2013). Loneliness Updated: Recent Research on Loneliness and How It Affects Our Lives.

[56] Rokach A., Heather B. (1997). Loneliness: A multidimensional experience. Psychol. J. Hum. Behav..

[57] Saunders P., Wong H., Wong W.P. (2014). Signposting disadvantage—Social exclusion in Hong Kong. J. Asian Public Policy.

[58] Buffel T., Phillipson C., Scharf T. (2013). Experiences of neighbourhood exclusion and inclusion among older people living in deprived inner-city areas in Belgium and England. Ageing Soc..

[59] Newall N. Social Isolation and Age-Friendly Communities. http://chnet-works.ca/images/stories/01_27_16_PPT_FINAL.pdf.

[60] Public Health Agency of Canada (2015). Age–Friendly Communities Evaluation Guide: Using Inicators to Measure Progress.

[61] Hong Kong Special Administrative Region Government Responding to Population Ageing: Building an Age-Friendly Community. http://www.policyaddress.gov.hk/2016/eng/pdf/PA2016.pdf.

[62] World Health Organization (2007). Global Age-Friendly Cities: A Guide.

[63] Pinquart M., Sorensen S. (2001). Influences on Loneliness in Older Adults: A Meta-Analysis. Basic Appl. Soc. Psychol..

[64] Bronfenbrenner U. (1994). Ecological models of human development. Read. Dev. Child..

[65] De Jong Gierveld J., Tesch-Römer C. (2012). Loneliness in old age in Eastern and Western European societies: Theoretical perspectives. Eur. J. Ageing.

[66] Hammersley M. (2006). Ethnography: Problems and prospects. Ethnogr. Educ..

[67] Maxwell J.A., Chmiel M., Flick U. (2014). Generalization in and from qualitative analysis. The Sage Handbook of Qualitative Research.

[68] Gibson H.B. (2000). The benefits of solitude. Loneliness in Later Life.

[69] Ettema E.J., Derksen L.D., van Leeuwen E. (2010). Existential loneliness and end-of-life care: A systematic review. Theor. Med. Bioeth..

[70] Elias N. (1985). Loneliness of the Dying.

[71] Sand L., Strang P. (2006). Existential loneliness in a palliative home care setting. J. Palliat. Med..

